# Preventive Intervention Program on the Outcomes of Very Preterm Infants and Caregivers: A Multicenter Randomized Controlled Trial

**DOI:** 10.3390/brainsci11050575

**Published:** 2021-04-29

**Authors:** Young-Ah Youn, Seung-Han Shin, Ee-Kyung Kim, Hye-Jeong Jin, Young-Hwa Jung, Ju-Sun Heo, Ji-Hyun Jeon, Joo-Hyun Park, In-Kyung Sung

**Affiliations:** 1 Department of Pediatrics, Seoul St. Mary’s Hospital, College of Medicine, The Catholic University of Korea, Seoul 06591, Korea; lea732@hanmail.net (Y.-A.Y.); sinky@catholic.ac.kr (I.-K.S.); 2 Department of Pediatrics, Seoul National University Children’s Hospital, Seoul 03080, Korea; revival421@empas.com (S.-H.S.); jhjgirl821004@hanmail.net (H.-J.J.); jyhtlcn@gmail.com (Y.-H.J.); 3 Department of Pediatrics, Seoul National University Bundang Hospital, Sungnam-Si 13620, Korea; 4 Department of Pediatrics, CHA Gangnam Medical Center, CHA University, Seoul 06135, Korea; wnslatjsanf@hanmail.net (J.-S.H.); g-daughter@hanmail.net (J.-H.J.); 5 Department of Pediatrics, Anam Hospital, Korea University College of Medicine, Seoul 02841, Korea; 6 Department of Rehabilitation Medicine, Seoul St. Mary’s Hospital, College of Medicine, The Catholic University of Korea, Seoul 06591, Korea; drpjh@catholic.ac.kr

**Keywords:** neurodevelopmental, primary outcome, preterm, very low birth weight, intervention, prevention

## Abstract

Increased survival in the very preterm population results in a higher risk of developing neurodevelopmental and behavioral disabilities among survivors. We examined the outcomes of very preterm infants and parents after a preventive intervention program of four home visits by a specialized nurse, 5 days, 2 weeks, and 1 month after discharge, respectively, and at CA 2 months, followed by up to 12 times of group sessions between CA 3 and 6 months. Our multicenter randomized controlled trial assessed 138 preterm infants (gestational age ≤30 weeks or birth weight ≤1500 g) enrolled from the three participating hospitals. We randomly allocated the preterm babies to either the intervention or the control group. The primary outcome was the neurodevelopmental outcomes of Bayley-III scores at CA 10 and 24 months. At CA 10 months and 24 months, there were no significant differences between the intervention and control groups in the cognitive, motor, and language domains of Bayley-III scores. In addition, there were no significant differences in the mother’s depression scale, mother–child attachment, and the modified Infant and Toddler Social and Emotional Assessment.

## 1. Introduction

Over the decades, there have been progressive advances in neonatal practice along with antenatal care, which has led to an increased survival rate of very preterm infants. Increased survival in this vulnerable population results in a higher risk of developing neurodevelopmental and behavioral disabilities among survivors [1]. After discharge from the intensive care unit, parents or primary caregivers play an irreplaceable role in the care and monitoring of very preterm babies [2]. The majority of parents of very preterm infants experience stress, depression, and anxiety, accompanied by a lack of confidence, which often results in poor parent–infant relationships [3,4,5,6]. This may result in adverse family functioning [4,5] with detrimental short- and long-term outcomes for their children [3,6]. Of the variety of early intervention programs tried, Kangaroo Care and the Mother–Infant Transaction Program have shown the most positive outcomes in infants [7].

Home visiting is a convenient and effective form of intervention immediately after discharge because the parents or caregivers are generally less confident and experience more anxieties from this lack of self-confidence in caring for their vulnerable preterm babies who were once cared intensively during the hospitalization. The preventive care program may offer improved maternal–infant interaction, more confident and happier parenting behavior, and more optimal home environments in preterm infants [8,9]. Among the programs, the group intervention has been proven to be helpful for parents of children with disabilities or abnormal behaviors [10,11] and has been included in the Infant Health and Development Program (IHDP) for preterm infants [12]. However, the appropriate timing and intensity of intervention to benefit the developmental capacity of preterm infants has not been established so far. Since anxiety and depression among the parents are the highest when they bring their preterm babies at home [5], an earlier and intense preventive care program might be beneficial for the post-discharge care of preterm infants. In our country, the improved survival of extremely preterm infants has resulted in a higher incidence of preterm babies discharged from the neonatal intensive care unit (NICU), but little family support is provided after discharge by the government or society. This results in anxiety and depression when they bring their preterm babies at home, resulting in a lack of maternal–infant interaction and worse neurodevelopmental outcomes [3,6,7]. Therefore, a family support program with timely interventions needs to be implemented for the very preterm babies who were cared for in the NICU.

The objective of this study was to examine the effects of an early preventive care program (consisting of 4 home visits and up to 12 group interventions at the center before the corrected age (CA) of 6 months) on the neurodevelopmental and behavioral outcomes in the infant. Additionally, maternal outcomes of depression or anxiety and mother–child attachment were also evaluated.

## 2. Methods

A multicenter randomized controlled trial was conducted in three tertiary hospitals in Seoul, Republic of Korea. These hospitals were members of the Follow-Up Task Force of the Korean Society of Neonatology. Infants who were born at gestational age ≤30 weeks or birth weight ≤1500 g were enrolled and randomized before discharge from the NICU to either the intervention group or a control group. As a data entry, each infant was randomly sequentially allocated to be either the control or intervention group by the computerized system of allocation (refer to Section 2.3). Infants with congenital neuromuscular diseases, cardiac anomalies, or chromosomal anomalies were excluded. Informed consent was obtained from the caregivers of the included children. The recruitment period was from May 2015 to July 2016, and the study was approved by the Ethics Committees of participating hospitals. All methods were carried out in accordance with relevant guidelines and regulations of the Ethics Committees of the participating hospitals. All subjects gave their informed consent before they participated in the study. The study was conducted in accordance with the Declaration of Helsinki by the Ethics Committees of Seoul National University Hospital (1501-097-642), Seoul St. Mary’s Hospital (KC15OINM0179) and CHA Gangnam Medical Center (2015-04-013-001). This trial has been registered at www.clinicaltrials.gov (identifier NCT02415530) (14/04/2015).

### 2.1. Intervention

**Figure 1:**
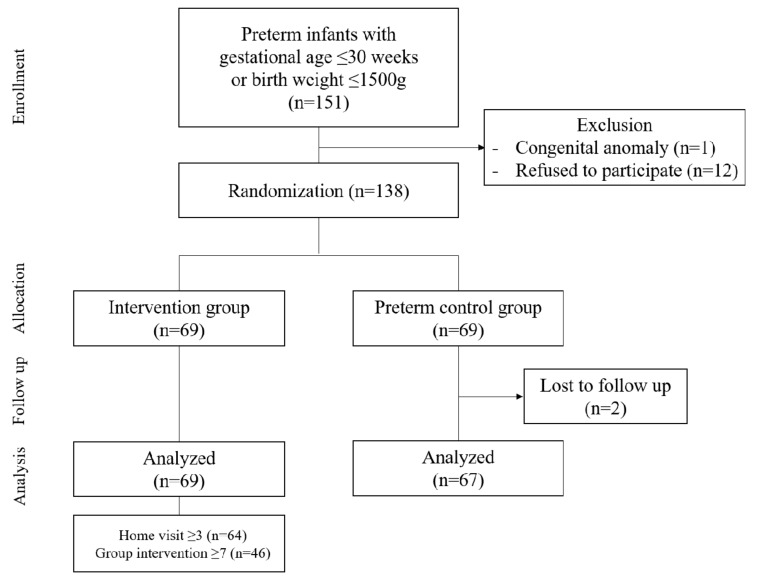
The CONSORT flow study diagram.

Initially, 151 patients were enrolled; 12 patients were excluded for their refusal to participate, and one patient was excluded due to congenital anomaly. The study flow is shown in Figure 1. As a result, a total of 138 infants were randomized (69 intervention and 69 preterm control groups). Infants in the control group received only standard care (no home visits or group interventions). The timing and periods of the home visits were set based on the Mother–Infant Transaction Program (MITP). The concept of group intervention was based on the IHDP but was introduced earlier than that of the IHDP [12]. Infants in the intervention group received home visits by nurses 5 days, 2 weeks, and 1 month, respectively, after discharge, and at 2 months of CA as a last visit. Fifteen nurses from the three participating hospitals were engaged in the home visits, and they were all experienced (≥5 years of NICU working experience) NICU nurse staff members. The purpose of the visit was to provide a better understanding of the baby’s behavioral cues, such as crying, temperament, satiety, and sleeping pattern, and support of the care of the baby on feeding, sleeping position, hygiene, defecation, and emergent situations. Checklists were used to standardize the procedure of evaluation and education of parents. From 3–6 months of CA, infants in the intervention group participated in the group intervention program up to 12 times with a physiotherapist specialized in infant neurodevelopment. This physiotherapist provided recommendations to the caregiver to enhance infant–parent bonding and approaches to promote the child’s growth and neurodevelopment. The specialized physiotherapist assisted by one experienced pediatric physiotherapist taught various activities to promote the infants’ gross motor development and sensory stimulation. Their main goal was to provide parental emotional support and to encourage attachment between parents and infants as well as to promote the infants’ gross motor development and sensory stimulation. In each activity, the infant’s behavior was carefully observed by parents and the care providers, after which parents were supervised to ensure their understanding of the infant’s regulatory efforts and to modify the environment according to the infant’s needs. Instructions on parent–infant interactions and postural control of the baby without distress were also given, and detailed information on the infant’s developmental milestones was explained to the parents. Each intervention group session, usually composed of 4–5 family members, lasted about 90 min and was conducted at a center in Seoul, Korea. During after-meeting congregations, parents were encouraged to share ideas and experiences.

### 2.2. Outcomes

The primary outcome of the study was the cognitive domain of Bayley Scales of Infant and Toddler Development—Revised III (Bayley-III) at CA 10 months. An experienced examiner in each of the two participating hospitals administered the Bayley-III, while the third hospital referred patients for the examination to one of the other two hospitals because they had no dedicated examiners. The examiners who conducted the Bayley scales were blinded to group allocations. Secondary outcomes included the mother-to-child attachment (MCA) score and the Center for Epidemiologic Studies Depression Scale (CES-D) [13] at CA 2 and 6 months, as well as the Infant Characteristics Questionnaire (ICQ) [14] at CA 10 months. At CA 24 months, Bayley-III, in addition to Korean Developmental Screening Test (K-DST), and the modified Infant and Toddler Social and Emotional Assessment (m-ITSEA) were evaluated.

The K-DST is a parent-reported developmental screening questionnaire developed by the Korean Pediatric Society [15]. This questionnaire encompasses gross motor, fine motor, cognition, language, personal, social, and self-help domains. The m-ITSEA was used to identify potential behavioral problems or delays in socioemotional competence by a screening questionnaire consisting of 82 items, derived from the original 169 items of the Infant and Toddler Social and Emotional Assessment [16]. Internal consistency was acceptable (α = 0.80 and 0.82, respectively), and good test–retest reliability has been demonstrated for the m-ITSEA with intraclass correlations of 0.82 for the problem scale and 0.72 for the competence scale.

A pre-specified questionnaire was used to obtain information about the socioeconomic status of the family. The questionnaire included information on marital status, monthly income of the family, and the primary caretaker of the baby. Age, education level, and type of job of parents were also collected. Demographics factors, such as gestational age, body size at birth, sex, mode of delivery, multiple birth, and Apgar scores, were obtained. Information on neonatal morbidities, such as respiratory distress syndrome, patent ductus arteriosus, sepsis, necrotizing enterocolitis, brain injury, bronchopulmonary dysplasia, duration of invasive ventilation, and retinopathy of prematurity, was also collected. Bronchopulmonary dysplasia and its severity were defined using the criteria from the National Institute of Child Health Workshop’s definition [17]. Retinopathy of prematurity (ROP) was staged according to the International Classification of ROP [18]. Necrotizing enterocolitis was defined as stage II or higher according to Bell’s Staging Criteria [19]. Papile classification was used to determine the grade of intraventricular hemorrhage (IVH) [20], and IVH with grade III or IV and periventricular leukomalacia was classified as severe brain injury.

### 2.3. Power Calculations and Randomization

The study size was calculated based on differences between the preterm intervention group and the preterm control group in the Bayley-III scores. With a group size of 70, there was an 80% chance to detect a difference between two groups of 10 in the cognitive score, with a level of significance of 0.05., anticipating 20% of follow-up loss in the study population. The infants were randomized sequentially using a web-based randomization program in a block design of size 2 and 4, which was run by the Medical Research Collaborating Center in Seoul National University Hospital. Random assignment was stratified according to singleton versus twin births and <28 weeks versus ≥28 weeks of gestational age. Children from multiple births were assigned randomly to the same group because the intervention was performed with a family-based approach.

### 2.4. Statistical Methods

Chi-square and Fisher’s exact tests were used for the analysis of categorical variables. The Wilcoxon rank-sum test was used to compare continuous variables between the preterm intervention group and the preterm comparison group. The Wilcoxon signed-rank test or the *t*-test were used to compare continuous variables. Values were expressed as *n* (%) or mean ± standard deviation.

## 3. Results

Of the 69 infants in the control group, 2 infants were lost to follow-up after discharge, resulting in a total of 67 infants. The mean gestational age and birthweight were comparable between the intervention and control groups (Table 1). In general, there were no significant differences in the demographic findings among the randomized very preterm infants. In addition, neonatal morbidities during the NICU hospitalization were not significantly different between the two preterm groups (Table 1). Severe brain injury such as intraventricular hemorrhage grade 3 or higher, parenchymal hemorrhage of the cerebrum or cerebellum, and periventricular leukomalacia were not significantly different between the groups. The duration of hospitalization and invasive ventilation were also comparable between the two groups. In most families, the primary caretakers were the mothers, and the distribution of income levels was similar in both very preterm groups (Table 2). Only one parent was not married at the time of enrollment. Other demographic data such as age, education level, income, and type of career were also not significantly different between the two groups.

As the outcome measures, there were no differences in the caregivers’ depression score noted in the CES-D score between the two groups at CA 2, 6, and 24 months (Table 4). Although the depression scores decreased in both groups as the infants grew older, there were no significant differences between the two groups in terms of changes in CES-D scores from baseline to CA 2, 6, and 24 months. Similarly, there were no significant differences in the MCA score at CA 2 and 6 months as well as changes in the MCAscores between the intervention and control groups. Additionally, the infants’ characteristics from the questionnaire at 10 months showed no significant improvement in the intervention group (Table 3). As a neurodevelopmental outcome, no significant differences were found in the Bayley-III at either CA 10 months or CA 24 months (Table 4). There were also no significant intergroup differences in the m-ITSEA and K-DST at CA 24 months.

## 4. Discussion

In the present study, our intervention program until CA 6 on very preterm babies did not have a significant effect on their neurodevelopmental outcomes.

The intervention group program was designed on the basis of the literature on infant development, parent mental health, the parent–infant relationship, and incorporated components of previously successful interventions [7]. Since the attribution to neurodevelopmental outcome might be weak for group intervention, we anticipated that the home visits and group intervention program might have a positive effect on the mother-to-child attachment score and less depression for caregivers. However, the attachment between mother and child and the mood of the mother or primary caregiver, in addition to infant characteristics, did not show any significant difference between the two groups.

Since the early 1980s, preventive care for preterm infants has been highlighted. The IHDP in the US was one of the earliest forms of preventive care programs developed at the time, and it consisted of home visits and child attendance at a child development center. A multisite, randomized trial reported that the IHDP improved IQ scores at 36 months of age and reduced behavioral problems in preterm infants [21]. The MITP, also developed in the 1980s, consisted of pre-discharge meetings and home visits and focused on the dynamic interplay between caregiver and child, helping caregivers understand the infants’ characteristics, temperament, and developmental potential [22]. Several randomized studies conducted in the 1990s also reported the benefits of early intervention on the cognitive function of preterm infants during infancy and preschool age [23,24]. A recent Cochrane review concluded that early intervention for preterm infants benefits both cognitive and motor functions during infancy and cognitive outcomes by preschool age [25]. However, many randomized studies, especially during the last two decades, have been unable to demonstrate the benefits of early interventions in preterm infants in cognitive outcomes [9,26,27,28] and motor outcomes [27,29] beyond one year of CA.

Rather, reduction in parental stress, anxiety, and depressive symptoms are commonly reported benefits from early interventions [8,9,30,31,32]. Moreover, the effects of early interventions on parent–infant interactions have been consistently reported [7,33,34]. Improvements in the temperaments and behaviors of preterm infants are reported to be the major later benefits of early interventions [35].

One of the reasons why our early intervention program failed to show a positive impact on the various outcomes may be because the length of intervention was too short or the assessment time was too short to manifest a significant effect. In our study, the participation of 12 whole-group interventions was low; only 46 children received more than 7 group sessions. The negative impact on cognitive and motor development in the preterm infants is in line with recent individual randomized trials [9,36]. However, the beneficial effects on parental anxiety, mother-and-child attachment, or behavioral outcomes of infants have been reproduced in many studies. Hence, the absence of observed benefits in the current study, especially in maternal anxiety and mother–infant attachment, indicates that the intervention was insufficiently effective for the families of preterm infants. Group interventions have been less frequently adopted for the preterm family compared to individual-basis interventions [7]. Parent-to-parent support in the group intervention can also elevate self-esteem or lessen the anxiety of parents by sharing personal experiences and tips in the better care of children. The length of appropriate interventions may influence the outcomes of preterm infants [9,37], and further studies are needed to be established. Finding suitable populations to benefit is another important issue in preventive care programs.

Following up on the study population until a more advanced age is necessary to determine the longer-term effects of this intervention as some interventions have shown delayed benefit. The study using the Infant Behavioral Assessment and Intervention Program (IBAIP) found that delay in the verbal and performance IQ is less frequent in the intervention group at 5.5 years of age, while there were no differences in the mental developmental index of the BSID-II and Behavior Rating Scale at 2 years of CA [38,39]. No significant effect on infant cognitive development was apparent until 36 months; however, the effect of the intervention became significant at 48 months in the MITP trial [22].

A limitation of the current study was that although most infants (92.7%) in the intervention group received home visits more than three times, only 66.7% of the intervention group received seven or more group interventions. This low compliance for group intervention might be due to center-based rather than home-based intervention, but analysis after excluding those who received group intervention less than seven times still showed no differences in the primary and secondary outcomes between the groups (data not shown). Another possible limitation could be that the measures used were designed to define developmental impairment/delay—it is possible that the preventative program impacted other factors that were not measured. In addition, our study contained a high number of multiples (38.8% in the intervention and 44.9% in the control group), meaning that family factors weighed heavily and washed out on analysis. Moreover, enrollment was closed prematurely before the number of participants reached 70 for each group, as the study was granted by a research fund with a fixed project period. However, follow-up rate was higher than we expected, and the number of participants included in the analysis was consequently higher than the required number in the sample size calculation. Lastly, restricting the trial to infants at highest risk for adverse neurodevelopment (i.e., <28 weeks of gestation) could have resulted in more substantial differences between groups. The assignment of twins to the same group may have diluted the randomization effect.

The strength of this study is a multicenter approach using an intention-to-treat analysis approach by evaluating a preventive program that comprises both home visits and group sessions to support child and caregiver outcomes.

## 5. Conclusions

The present study showed that an intervention program for very preterm infants provided intensively before CA 6 months with home visits and the subsequent group at a specialized developmental care center showed no significant positive effect on neurodevelopmental outcomes of very preterm infants, nor on maternal anxiety, mother–child attachment, and behaviors. Evidence-based intervention is crucial to effectively use social resources. Reassessment of the children and their families at a later age is necessary for determining the longer-term benefits of this program. Nevertheless, the families of very preterm infants are obviously in need of support as these infants are at a high risk for developmental problems and the parents suffer from enormous emotional burdens. Further studies with longer duration and effective modalities for outcome measures are warranted to find beneficial intervention programs for very preterm infants as a nationwide implementation.

## Figures and Tables

**Table 1:** Clinical characteristics and outcomes of very preterm babies: intervention vs. control (*n* = 136).

	Intervention (*n* = 69)	Control (*n* = 67)	*p*-Value
GA (week) ^§^	29 ± 2.6	29 ± 2.5	0.995
Birth weight (g) _§_	1145.5 ± 344.5	1188.9 ± 340.6	0.758
Birth height (cm) ^§^	37.4 ± 3.8	37.3 ± 4.5	0.995
Birth HC (cm) ^§^	26.5 ± 2.8	26.7 ± 2.9	0.884
Female ^¶^	38 (56.7)	31 (44.9)	0.176
Antenatal steroid ^¶^	38 (55.9)	33 (51.6)	0.727
hCAM ^¶^	21 (35)	16 (29.6)	0.556
C/S ^¶^	52 (77.6)	47 (69.1)	0.331
Multiple birth ^¶^	26 (38.8)	31 (44.9)	0.492
AS 1 min ^§^	3.7 ± 2	4.1 ± 1.9	0.417
AS 5 min ^§^	6.1 ± 2	6.4 ± 1.8	0.663
Cord ABGA pH ^§^	7.3 ± 0.1	7.3 ± 0.1	0.770
RDS ^¶^	54 (78.3)	57 (85.1)	0.378
Treated PDA ^¶^	18 (26.5)	23 (34.9)	0.350
Sepsis ^¶^	9 (13)	12 (18.5)	0.478
NEC ^¶^	12 (17.4)	7 (10.5)	0.324
Brain injury			0.559
low-grade IVH ^¶^	26 (37.7)	21 (31.3)	
severe injury ^¶^	9 (13)	13 (19.4)	
Moderate to severe BPD ^¶^	18 (26.1)	21 (31.8)	0.569
Steroid for BPD ^¶^	15 (21.7)	19 (28.4)	0.431
Invasive ventilation (d) §	16.9 ± 30.9	16.8 ± 24.4	0.353
ROP ^¶^	32 (46.4)	40 (59.7)	0.127
ROP requiring operation ^¶^	4 (7.3)	12 (10.5)	0.365
Hospital stay (d) ^§^	68.3 ± 36.4	64.9 ± 32.2	0.652
PMA at discharge (week) ^§^	38.7 ± 3.4	38.6 ± 3.1	0.919
Weight at discharge (g) ^§^	2644.8 ± 681.3	2686.8 ± 643.4	0.550
Height at discharge (cm) ^§^	45.2 ± 3.5	45.1 ± 3	0.902
HC at discharge (cm) ^§^	32.3 ± 2	32.3 ± 2.3	0.836

The values are expressed as *n* (%) ^¶^ or mean ± SD ^§^. Abbreviations: GA, gestational age; HC, head circumference; hCAM, histologic chorioamnionitis; C/S, Cesarean section; AS, Apgar score; ABGA, arterial blood gas analysis; RDS, respiratory distress syndrome; PDA, patent ductus arteriosus; NEC, necrotizing enterocolitis; IVH, intraventricular hemorrhage; BPD, bronchopulmonary dysplasia; ROP, retinopathy of prematurity; PMA, postmenstrual age; HC, head circumference.

**Table 2:** Socioeconomic characteristics of very preterm infants.

	Intervention (*n* = 69)	Control (*n* = 67)	*p*-Value
Marriage	69 (100)	66 (98.5)	0.493
Income (thousand won)			0.491
<2000	2 (2.9)	3 (4.5)	
2000~4000	30 (43.5)	30 (44.8)	
>4000	37 (53.6)	34 (50.8)	
Primary caretaker—mother	66 (95.7)	65 (97)	1.000
Information on father			
Age (year)	37 ± 4.9	37 ± 3.5	0.740
College education or above	59 (85.5)	63 (94)	0.157
Job			0.667
salaried employee	41 (59.4)	41 (62.1)	
public officials	1 (1.5)	4 (6.1)	
self-employed	8 (11.6)	7 (10.6)	
professional	14 (20.3)	10 (15.2)	
others	5 (7.3)	4 (6.1)	
Information on mother			
Age (year)	34.8 ± 4.2	34.1 ± 2.9	0.296
College education or above	59 (85.5)	61 (91)	0.556
Job			0.472
salaried employee	19 (27.5)	14 (20.9)	
public officials	1 (1.5)	0 (0)	
self-employed	1 (1.5)	3 (4.5)	
professional	9 (13)	6 (9)	
others	39 (56.5)	44 (65.7)	

**Table 3:** CES-D, mother-to-child attachment score, and ICQ.

	Intervention (*n* = 69)	Control (*n* = 67)	*p*-Value
CES-D			
baseline	11 ± 7.7	13.4 ± 9.2	0.263
2 months after discharge	9 ± 7.1	10.3 ± 6.5	0.690
6 months after discharge	7.5 ± 6.3	8.8 ± 6.8	0.615
24 months after discharge	9.3 ± 7.9	8.1 ± 7.1	0.744
Δ baseline—2 months after discharge	−1.7 ± 7.5	−3 ± 9.7	0.700
Δ baseline—6 months after discharge	−3.5 ± 6.3	−4.6 ± 10.1	0.730
Δ baseline—24 months after discharge	−1.4 ± 7.9	−4.5 ± 10.8	0.217
Mother-to-child attachment score			
2 months after discharge	99.3 ± 4.7	99.6 ± 5.5	0.973
6 months after discharge	100.1 ± 4.3	101.3 ± 5.1	0.348
Δ 2 months after discharge—6 months	0.6 ± 3.9	1.8 ± 4	0.420
ICQ at CA 10 months			
Fussy	30.3 ± 6.5	27.5 ± 7.3	0.071
Unadaptable	14.8 ± 4.4	14.9 ± 4.4	0.989
Dull	13 ± 2.2	12.5 ± 3	0.501
Unpredictable	18 ± 4.5	17.5 ± 3.9	0.755
Total score	74.9 ± 16	72 ± 13.7	0.545

The values are expressed as mean ± SD. Δ means change of score from baseline to each time point. CES-D, Center for Epidemiologic Studies Depression Scale; MCA, mother-to-child attachment; ICQ, Infant Characteristics Questionnaire; CA, corrected age.

**Table 4:** Neurodevelopmental outcomes of very preterm babies: intervention vs. control.

	Intervention (*n* = 69)	Control (*n* = 67)	*p*-Value
BSID 10 months (*n*)	66	61	
Cog _§_	99.5 ± 10.8	98.4 ± 11.7	0.834
Lang ^§^	92.8 ± 8.3	92.7 ± 10.1	0.996
Motor ^§^	93.7 ± 12.6	93.9 ± 12.8	0.997
BSID 24 months (*n*)	43	39	
Cog ^§^	100.3 ± 17.1	98.3 ± 15.4	0.779
Lang ^§^	95.3 ± 17.1	94.6 ± 17.2	0.727
Motor ^§^	95 ± 16.9	96.3 ± 19.1	0.586
Modified BITSEA (*n*)	61	53	
Externalizing ^§^	11.6 ± 5.5	10.6 ± 4.1	0.579
Internalizing ^§^	12.6 ± 5.1	11.9 ± 4.2	0.741
Competence ^§^	48.7 ± 11.9	52.5 ± 12.2	0.210
K-DST	41	38	
Gross motor ^§^	19.3 ± 4.5	18.3 ± 6.6	0.645
Fine motor ^§^	18.6 ± 4.1	18.8 ± 3.7	0.964
Cognitive ^§^	17.3 ± 5.8	19.1 ± 4.2	0.232
Language ^§^	16.3 ± 7.6	15.8 ± 7.5	0.936
Personal Social ^§^	17.6 ± 5.8	17.7 ± 5.5	0.996
Self-help	19.1 ± 4.3	18.2 ± 5.9	0.630
Gross motor < cut-off ^¶^	7 (11.9)	5 (9.4)	0.766
Fine motor < cut-off ^¶^	4 (6.8)	3 (5.7)	1.000
Cognitive < cut-off ^¶^	6 (10.2)	6 (11.5)	1.000
Language < cut-off ^¶^	6 (10.2)	3 (5.7)	0.495
Personal Social < cut-off ^¶^	3 (5.1)	2 (3.8)	1.000
Self-help < cut-off ^¶^	3 (5.1)	6 (11.3)	0.303

The values are expressed as *n* (%) ^¶^ or mean ± SD ^§^. Abbreviations: M-ITSEA, modified Infant and Toddler Social and Emotional Assessment; K-DST, Korean Developmental Screening Test.

## Data Availability

The datasets generated and analyzed are not publicly available but are available from the corresponding author on reasonable request.

## Abbreviations

Bayley-III	Bayley Scales of Infant Development-Revised III
CA	corrected age
CES-D	Center for Epidemiologic Studies Depression Scale
GA	gestational age
IBAIP	Infant Behavioral Assessment and Intervention Program
ICQ	Infant Characteristics Questionnaire
IHDP	Infant Health and Development Program
IQ	intelligence quotient
K-DST	Korean Developmental Screening Test
MCA	mother-to-child attachment
MITP	Mother–Infant Transaction Program
m-ITSEA	modified Infant and Toddler Social and Emotional Assessment
NICU	neonatal intensive care unit
PMA	postmenstrual age
